# Changes in mental health services in response to the COVID-19 pandemic in high-income countries: a rapid review

**DOI:** 10.1186/s12888-024-05497-6

**Published:** 2024-02-06

**Authors:** Evgenia Stepanova, Alex Thompson, Ge Yu, Yu Fu

**Affiliations:** 1 Population Health Sciences Institute, Newcastle University, Newcastle, UK; 2 Health Services and Population Research Department, Institute of Psychiatry, King’s Health Economics, King’s College London, Psychology & Neuroscience, London, UK; 3 Department of Primary Care and Mental Health, Institute of Population Health, University of Liverpool, Liverpool, UK

**Keywords:** Mental Health, COVID-19, Telehealth

## Abstract

**Background:**

Severe deterioration in mental health and disrupted care provision during the COVID-19 increased unmet needs for mental health. This review aimed to identify changes in mental health services for patients in response to the pandemic and understand the impact of the changes on patients and providers.

**Methods:**

Following the Cochrane guidance for rapid reviews, Cochrane CENTRAL, MEDLINE, Embase and PsycInfo were searched for empirical studies that investigated models of care, services, initiatives or programmes developed/evolved for patients receiving mental health care during COVID-19, published in English and undertaken in high-income countries. Thematic analysis was conducted to describe the changes and an effect direction plot was used to show impact on outcomes.

**Results:**

33 of 6969 records identified were included reporting on patients’ experiences (*n* = 24), care providers’ experiences (*n* = 7) and mixed of both (*n* = 2). Changes reported included technology-based care delivery, accessibility, flexibility, remote diagnostics and evaluation, privacy, safety and operating hours of service provision. These changes had impacts on: (1) care access; (2) satisfaction with telehealth; (3) comparability of telehealth with face-to-face care; (4) treatment effectiveness; (5) continuity of care; (6) relationships between patients and care providers; (7) remote detection and diagnostics in patients; (8) privacy; (9) treatment length and (10) work-life balance.

**Conclusions:**

A shift to telecommunication technologies had a significant impact on patients and care providers’ experiences of mental health care. Improvements to care access, flexibility, remote forms of care delivery and lengths of operating service hours emerged as crucial changes, which supported accessibility to mental health services, increased attendance and reduced dropouts from care. The relationships between patients and care providers were influenced by service changes and were vastly depending on technological literacy and context of patients and availability and care access ranging from regular contact to a loss of in-person contact. The review also identified an increase in care inequality and a feeling of being disconnected among marginalised groups including homeless people, veterans and ethic minority groups. Telehealth in mental care could be a viable alternative to face-to-face service delivery with effective treatment outcomes. Further research is needed to better understand the impact of the changes identified particularly on underserved populations.

**Supplementary Information:**

The online version contains supplementary material available at 10.1186/s12888-024-05497-6.

## Background

The outbreak of Covid-19 resulted in the implementation of extensive infection control measures worldwide. National and subsequent local lockdown measures were imposed by the UK government during which the public was instructed to stay at home, socially distance and self-isolate with strict guidance about movement outside one’s household [1]. During this period of restrictions and beyond, there was a severe deterioration in mental health [2, 3]. The prevalence of mental health symptoms increased in previously healthy people with an additional 5.8% of adults in the UK reporting clinically significant levels of psychological distress [4] and increases in depression, anxiety and traumatic stress [5]. Individuals already experiencing mental health issues reported worsening symptoms with 65% of adults with mental health conditions surveyed reporting that their mental health deteriorated during this time [6, 7]. It was predicted that the mental health impact of the pandemic and associated lockdown measures would lead to additional mental health support needs for 10 million individuals in the UK, around 20% of the population which was expected to exceed the capacity of the NHS by 2–3 times in a 3–5 year window [8].

This increase in demand for essential mental health services was coupled with severe disruption to mental health care provision [9, 10]. To abide by infection control measures, mental health service providers were required to adapt their service provision [6]. Many service providers made changes such as ceasing or reducing face-to-face appointments, offering remote treatment sessions and altering their operating hours [9]. These alterations to care were recognised as having the potential to be disproportionately detrimental to those living with mental health conditions prior to the pandemic as well as those with newly developed mental health issues [11]. Difficulties attending review appointments in person and closure of support services were likely to impact all those in, or in need of, active treatment [12]. For example, those with worsening pre-existing mental health conditions who encountered poor access to services reported experiencing relapse and suicidal behaviour [6] and adults with mental health conditions identified that disruptions to health services were impacting their mental health trajectory [13].

The consequences of lockdown restrictions on those with pre-existing mental health conditions were further compounded as these individuals experienced disproportionately worse mental health during the pandemic [13]. This group were more likely to report steady deterioration of their mental health or very poor mental health compared to adults without existing mental health conditions [14]. Similarly, people with severe mental health illnesses faced significant health inequalities which were exacerbated and further entrenched by the unequal impacts of COVID-19 such as digital exclusion [15].

In addition to this, mental health services failed to meet the increased demand for support and treatment during this time, increasing the mental health prevention and care provision gap that existed prior to the pandemic [16].

The UK National Health Service (NHS) has set up a long-term plan to address this gap in mental health care service provision which is widely regarded as being under-resourced [17]. The COVID-19 pandemic has increased awareness of mental health issues, further highlighted these mental health service inadequacies, and has altered mental health service provision [6, 18]. As such, the pandemic offers an opportunity to rethink conventional approaches to mental health services. Learning from service changes throughout the pandemic and the consequences for care recipients and providers is vital to inform practical policy solutions for integrated service recovery and effectively plan services that reach those with the greatest need.

To date, no review has synthesised the available evidence relating to these service alterations for adult patients. As such, the overall aim of this review is to: (1) identify changes in mental health service provision for adult patients in response to the pandemic and (2) understand the impact of the changes on both patients and service providers.

## Method

This rapid review (protocol published [19], PROSPERO registration number CRD42022306923) is part of a larger mixed method study (protocol published [20] aiming to establish culturally competent mental health services which also consists of an observational study of routinely collected primary and secondary care data, qualitative interviews with service users from ethnic minority backgrounds, and a Delphi study to establish consensus on core service provision.

The rapid review was guided by the Cochrane guidance for rapid review (Cochrane, 2020) PRISMA statement [21]. Feedback and comments were actively sought from the project advisory group who met twice a year to inform the design and delivery of this review. Patient and public members helped identify the key terms and phrases used in the search strategy and were presented with steps undertaken and preliminary findings.

### Eligibility criteria

Studies were eligible if they reported operational changes in mental health services such as new models of care/services, initiatives, adaption/expansion of existing services or changes in service delivery model in response to COVID-19 to provide support for patients aged 18 and over, published in the English language from January 2019 to present. All empirical studies, regardless of study design, conducted in an Organisation for Economic Co-operation and Development (OECD) country [22] (to ensure a degree of commonality in health system and socioeconomic and demographic context) were eligible for inclusion. We did not include grey literature, editorial commentaries, protocols or conference abstracts, views of the general public and letter of opinion to peer-review journals.

### Search strategy

We searched four electronic databases including Cochrane CENTRAL, MEDLINE, Embase and PsycInfo using a range of keywords and subject headings validated by the information specialist representing COVID-19, mental health and OECD countries described in a Supplementary file 1. Searches were carried out in August 2022.

### Selection process

All records were exported to Rayyan [23], an online tool for review screening, for deduplication. All were screened by two reviewers (ES, AT) by title first followed by abstract if unsure. There was a 98% agreement rate. Where it was unclear based on title and abstract, the full texts were retrieved. Following the screening of titles and abstracts, full papers were retrieved and assessed for inclusion independently against the eligibility criteria by ES and AT. Where there was disagreement or uncertainty, studies were retrieved through consensus discussion with the research team.

### Data extraction

Data extraction was carried out using two forms: one developed for mental health patients and one for care providers. ES and AT independently piloted the data extraction forms on a sample of four studies with different study designs. Extracted data included geographical location; population group; study design, methodology; description of mental health service provision prior to COVID-19; changes in service during COVID-19; experiences of service change and impacts and outcomes reported.

All remaining data extraction was conducted by ES and verified and appraised by the team through regular reflexive meetings. Studies included were scrutinised to ensure all relevant data were captured and extracted as appropriate. The review process did not incorporate an assessment of risk of bias as per protocol [19]. To ensure quality assessment a tabulated and narrative synthesis was conducted. This approach allowed to report and discuss the results of the included studies in accordance with best practice guidance [24–26].

### Data synthesis

Guided by transparent and reproducible evidence synthesis we selected thematic and tabulated analysis of the data [24, 25, 27]. First, thematic analysis was selected to allow considerable latitude to reviewers and enabled the analysis of findings from both qualitative, quantitative and mixed methodologies [28]. Using this approach, we familiarised ourselves with the completed data extraction forms and inductively generated themes. Thematic analysis of each study was carried out to sort the findings into conceptual categories and groups.

Secondly, findings from the included studies were synthesised using tables and a narrative summary to report changes identified and their impacts reported as per protocol following current best practice to conduct synthesis systematically and transparently [24, 25, 29]. Meta-analysis was not possible because the included studies were heterogeneous in terms of the populations, study designs, methods and outcomes reported. Instead, we used effect direction following the Cochrane guidance [30] which allowed us to tabulate the reported heterogeneous changes in services and access the complex set of multiple outcomes. We applied the developed effect direction plot method with some adaptation to the characteristics of our research [30, 31]. A visual representation with arrows demonstrated the effect direction reported where ▲ was seen as an improvement in the outcome; ▼ was a deterioration in outcome and ◀▶ was no change. Due to a variety of study designs included, pooled analysis was not deemed appropriate, therefore we did not indicate study size and statistical significance.

## Results

### Search results

**Fig. 1:**
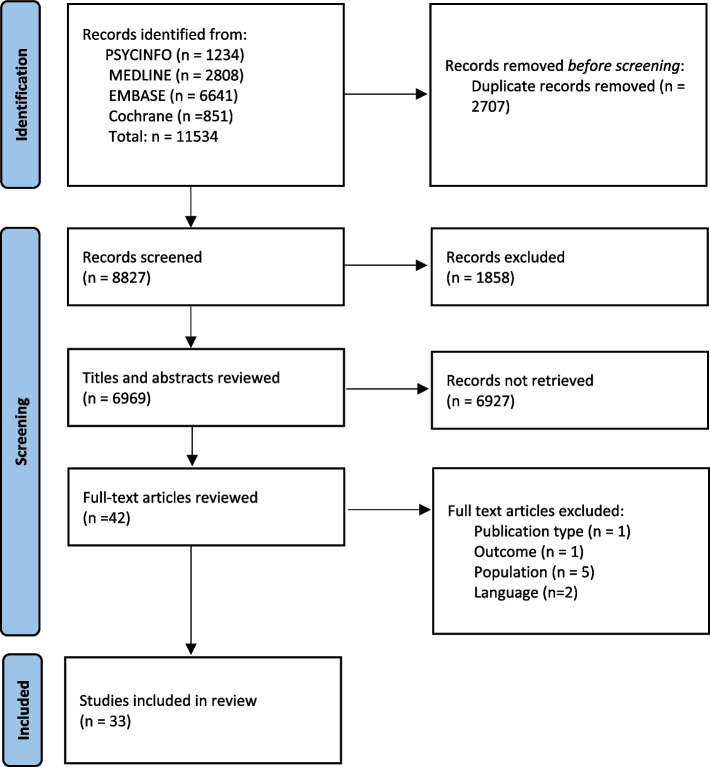
PRISMA diagram demonstrating search strategy

A total of 6969 records were screened and 42 were assessed potentially eligible based on title and abstract. Of these, 33 studies met the inclusion criteria and were included in the review. A PRISMA flow chart is presented in Fig. 1.

### Study characteristics

Of the 33 studies included, 13 were quantitative, 12 were qualitative, 5 reported case studies, 2 were RCT, 2 were descriptive and 1 used mixed methods. The majority of studies focused on mental health patients (*n* = 24) followed by mental health service providers (*n* = 7) or both (*n* = 2). Nineteen of the studies were conducted in the US, and the rest were in the UK (*n* = 4), EU countries (*n* = 6, Ireland (*n* = 2), Italy (*n* = 1), Spain (*n* = 1), Austria (*n* = 1) and the Netherlands (*n* = 1), Canada (*n* = 3) and Japan (*n* = 1). Table 1 provides an overview of the included studies.

**Table 1:** Study characteristics table

**ID**	**Author**	**Study Design**	**Country**	**Participants**	**Type of service delivery**	**Service change**	Abstract of Outcomes
**Mental health patients**							
1	Abate 2021 [31]	Case study	Italy	People with pre-existing diagnosed psychiatric disorders, especially severe or complex ones	Telephone	Remote diagnostics, accessibility of services, flexibility for services, care delivery mode via technology	Reduced burden on hospital care providers; effective and accessible services; challenges in assessment including difficulty in understanding patient's stress symptoms and general condition; difficulties with establishing trust between a specialist and a user
2	Adams 2022 [32]	Qualitative	UK	Homeless people, lived experiences of substance use and mental health illness	Telephone/videoconferencing	Operating hours of service provision, accessibility to services, privacy of care providers and patients, flexibility of services, care delivery mode via technology	a) reduced service provision, within working hours; b) negative experiences of participants as they could not access services when needed (prior to COVID negative experience)
3	Agyapong 2021 [33]	RCT	Canada	General population	Phone text service	Accessibility to services	This study demonstrated the effectiveness of Text4Hope over six consecutive weeks on various psychological symptomatology, including stress, GAD, MDD, and suicidal ideation or thoughts of self-harm, but not for disturbed sleep symptoms; statistically significant reductions in the prevalence rates for clinically meaningful stress, anxiety and depression as well as statistically significant reductions in mean scores on the PSS-10, GAD-7, and PHQ-9 scales when comparing the baseline and third month assessments in subscribers of Text4Hope)
4	Avalone 2021 [34]	Quantitative	US	Mental health patients	Telephone	Accessibility to services, flexibility of services, care delivery mode via technology	a) Higher completion rates in telehealth: outpatient adult mental health clinic telepsychiatry appointments, largely by telephone, were strongly associated with a higher rate of visit completion compared with in-person visits during and prior to the COVID-19 pandemic
5	Abdullah 2021 [35]	Qualitative	US	Patients with psychiatric disorders	Telephone	Accessibility to services, privacy of care providers and patients, flexibility of services, care delivery mode via technology	the experiences of both patients and service providers are mixed. Positive include flexibility, low drop-out rate, accessibility. Negatives include lack of privacy for patients, technology problems and care providers isolation
6	Barry 2022 [36]	Qualitative	Ireland	Patients with various mental health illness	Face to face /phone	Operating hours of service provision, accessibility to services, flexibility of services	a) flexibility and b) clarity in service provision pathways
7	Bean 2022 [37]	Quantitative	US	Patients seeking treatment for co‐occurring mental health and substance use disorder diagnoses	Videoconferencing	Accessibility to services, remote diagnostics and evaluation, flexibility of services, care delivery mode via technology	a) both services are effective in reducing symptoms of depression, anxiety, and stress when delivered in either in‐person or videoconference formats, with no significant difference between the forms of delivery in terms of symptom improvement
8	Bulkes 2022 [38]	Quantitative	US	Adults with anxiety and depressive symptoms	Videoconferencing	Remote diagnostics and evaluation, accessibility to services, flexibility of services	a) Increased length of stay in treatment in telehealth, b) remote treatment as a viable alternative to in-person mental health services, specifically as both in-person and remote patients experienced symptom reduction, and both populations reported improvements in quality of life
9	Burton 2021 [39]	Qualitative	US	Prison inmates with psychiatric disorders	Videoconferencing	Operating hours of service provision, remote diagnostics and evaluation, accessibility of services	a) high overall satisfaction with telepsychiatry so far; b) care providers have noted that they are often able to provide similar quality of services to patients over video as they are in person, and c) patients appreciate being able to meet with clinicians without coming into close contact with others. d) Clinics seem to run efficiently, as telepsychiatry reduces the time required for custody care providers to escort patients from housing units to treatment areas. e) Waiting rooms appear less congested than before
10	Gannon 2021 [40]	Case study	US	Distinct clinical populations, including general child and adolescent, intellectual and developmental disability, geriatric, general adult, addiction medicine, and psychotic disorders	Videoconferencing	Remote diagnostics and evaluation, accessibility to services, privacy of care providers and patients, flexibility of services, care delivery mode via technology	a) attendance increased; b) telehealth is a welcomed alternative; c) patients with anxiety, low mood, trauma, memory impairment, or psychotic spectrum disorders found telehealth less stressful d) for care providers seeing home environment of patients is beneficial (real-time observations) e) mobility of care providers allowed more flexibility and availability f) billing procedures are challenging; g) technology issues: lack of adequate hardware from care providers; h) privacy concerns of care providers
11	Glancy 2020 [41]	Qualitative	Ireland	Individuals with severe chronic and enduring mental health illnesses	Face to face/ videoconferencing	Care delivery mode via technology	a) physical isolation helped patients to have more space for reflection; b) self-awareness improved; c) sense of camaraderie was created
12	Guinart 2020 [42]	Quantitative	US	Patients in psychiatric centres	Videoconferencing	Remote diagnostics and evaluation, privacy of care providers and patients, flexibility of services, care delivery mode via technology	a) high levels of satisfaction with telepsychiatry services; b) the option of telepsychiatry should remain tailored to individual patient needs and be the result of shared decision; c) subjects were more likely to strongly agree to consider using telepsychiatry in the future when using video; d) lack of closeness; e) fear of reduction in the doctor’s ability to detect subtle signs of body language, nonverbal cues, and/or physical signs of disease could be some of the reasons behind this preference
13	Haderlein 2022 [43]	Qualitative	US	VA Primary Care-Mental Health Integration patients: veterans	Electronic consults, video consultations and messaging via the electronic health record	Accessibility to services	a) veterans who attended an initial PC-MHI mental health visit via telehealth were less likely to receive same-day primary care compared to veterans who initiated care in person; b) White veterans, and Hispanic veterans were more represented among telehealth patients than in-person patients, while women and Black veterans were less represented among telehealth patients than in-person patients; c) the PC-MHI model is designed to serve as an entry point into mental health services, with the intent to increase patient access to VA mental health care
14	Juan 2021 [44]	Qualitative	UK	People with pre-existing mental health conditions	Telephone and videoconferencing	Remote diagnostics and evaluation, accessibility to services, care delivery mode via technology	a) patients appreciated remote care options during the height of the pandemic when other forms of care were not possible; b) remote care was mainly seen as an option to allow access to care in extreme circumstances, rather than an alternative of comparable quality to face-to-face care; c) Other variables influencing remote care experiences were the relationship with the care provider, including whether they had met face-to-face in the past, and ease of use or access to necessary technology; f) Overall, participants stressed the need to provide alternatives for people who could not access or did not feel comfortable with telemental healthcare
15	Milosevic 2022 [45]	Quantitative	Canada	Outpatients of a tertiary care anxiety disorders clinic who attended a CBT group for panic disorder/ agoraphobia, social anxiety disorder, generalized anxiety disorder (GAD), or obsessive–compulsive disorder	Videoconferencing	Care delivery mode via technology	a) Significantly more sessions were attended by participants in the videoconference versus face-to-face GAD groups; b) Treatment dropout did not differ significantly between groups; c) a small but significant positive effect of face-to-face treatment on reduction in symptom severity over time, relative to videoconference treatment ( only the GAD group showed greater symptom improvement in the face-to-face format); d) Effect sizes (Cohen’s d) for treatment were mostly comparable between face-to-face and videoconference delivery, with videoconferencing tending to have slightly lower effects than face-to-face e) improved functional impairment over the course of treatment
16	Molino 2022 [46]	Case study	US	Patients with social anxiety disorder	Videoconferencing	Accessibility to services, flexibility of services	a) possibility that a transition to CBT via telehealth affected the potential trajectory of progress: symptom measure scores decreased; b) the telehealth helped to address acute stressors as they arose; c) the telehealth provided the patient with an opportunity to start attending support group as she did not need to physically travel to the clinic
17	Pinciotti 2022 [47]	Quantitative	US	Veterans with obsessive–compulsive disorder (OCD)	Videoconferencing, internet-based CBT (recorded sessions)	Accessibility to services, care delivery mode via technology	a) telehealth improved access to specialized mental health services for some individuals who may otherwise have been unable to access them
18	Puspitasari 2021 [48]	Qualitative	US	Patients with serious mental illness (adults living with an SMI, such as bipolar disorder or recurrent major depression, are at increased risk for substance abuse, homelessness, and death by suicide)	Videoconferencing	Remote diagnostics and evaluation, care delivery mode via technology	demonstrated the feasibility and initial effectiveness of ATP, a program that was rapidly switched to a video teleconferencing format during the COVID-19 pandemic. a) the completion rate was higher than typical completion rates for psychiatric IOP or PHP programs b)the average number of days completed by patients was 14.43 (SD 1.22), which indicated that the majority of patients only missed approximately 1 day in the three-week program c) patients' symptoms improved from admission to discharge; d) both the shared and differing content across the tracks were similarly effective in reducing distress and improving quality of life
19	Roncero 2020 [49]	Qualitative	Spain	Patients, service providers of The Salamanca Psychiatry Department (PS)	Telephone	Remote diagnostics and evaluation, accessibility to services, operating hours of service provision	a) Psychiatry service was carried out in three main aspects: generalizedimplementation of telemedicine, physical shutdown of the resources, and reorganization of human resources with those professionals who were not off work due to COVID nor were included in the COVID teams. b) The usage of tele-medicine in an extensive way, with around 9000 calls in 8 weeks, was successful in all the resources; c) patients have remained stable and their subjective perception of the support given was hardly lesser than with conventional hospitalization; d) accessibility: telemedicine can be a very relevant resource in the attention to geographically distant patients, urgent and pre-emptive
20	Saunders and Allen, 2021 [50]	Case study	UK	For adults with chronic physical health problems and either persistent subthreshold depressive symptoms or mild to moderate depression	Telephone	Accessibility to services	a) maintaining therapeutic homework was a challenge; b) monitoring behavioural work over the phone was difficult; c) The F2F sessions had focused on setting up behavioural goals, many of which could not be completed due to the pandemic, so resetting these goals led to some repeated work. The cognitive work on the other hand, worked well over the phone and was largely unchanged by phone delivery; d) Access: A move to telephone delivery meant sessions were still accessible. c) despite the change in modality, sessions were also able to remain largely reminiscent of how they had been during F2F delivery. (For example, email was utilized to deliver worksheets before and after sessions and this allowed AB to follow along with the CBT in a similar manner to how she had in F2F sessions.) e) digital divide between populations
21	Skime 2022 [51]	Quantitative	US	Adults with SMI who were recently discharged from psychiatric hospitalization or were at risk of psychiatric hospitalization	Videoconferencing	Accessibility to services, care delivery mode via technology	a) Patients were satisfied with the TMH ATP, and IOP, with most reporting that they would recommend these services to a friend or family member; b) “hybrid” model of care, which allows for both approaches (depending upon the patient’s choice and availability of stable internet services in their area) may be a viable alternative; c) TMH services are important in reaching patients that are geographically distanced from mental health facilities
22	Yahara 2021 [52]	Case study	Japan	Patients with mild cognitive impairment (MCI)	iVR reminiscence session	Accessibility to services, care delivery mode via technology	a) iVR reminiscence session may transiently reduce anxiety in the late elderly with MCI without causing serious side effects, which may also reduce the burden of caregiving for their families; b) the effectiveness of remote iVR reminiscence may be comparable to that of face-to-face iVR reminiscence; c) decrease in STAI scores after remote iVR reminiscence session, and his satisfaction level was higher than that of the final face-to-face iVR reminiscence session
23	Zimmerman 2021 [53]	Quantitative	US	Acute psychiatric patients who require a higher level of care	Videoconferencing	Remote diagnostics and evaluation, accessibility to services, flexibility of services	a) delivering treatment using a virtual, telehealth platform was as effective as treating patients in person; b) patients were satisfied with the initial diagnostic evaluation and were optimistic at admission that treatment would be helpful; c) Both treatment groups reported a significant reduction in symptoms and suicidality from admission to discharge and reported a significant improvement in functioning, coping ability, positive mental health, and general well-being; d) a greater length of stay and greater likelihood of staying in treatment until completion in the virtually treated patients; e) attendance rates: a lower “no show” rate for telehealth visits during the pandemic compared to in-person visits; e) treatment completion rate was significantly higher in the telehealth cohort
24	Zimmerman 2022 [54]	Mixed-method	US	Patients with psychiatric disorder	Videoconferencing	Remote diagnostics and evaluation, accessibility to services, flexibility of services	a) telehealth platform was as effective as treating patients in-person; b) patients were satisfied with the initial diagnostic evaluation and were optimistic at admission that treatment would be helpful; c) Both treatment groups reported a significant reduction in symptoms from admission to discharge, and both groups reported a significant improvement in functioning, coping ability, positive mental health, and general well-being; d) a slightly higher proportion of patients completed treatment in the telehealth program e) a greater length of stay and greater likelihood of staying in treatment until completion in the virtually treated patients
**Mental health care providers**							
25	Liberati 2021 [55]	Qualitative	UK	Adults with mental health difficulties under the care of secondary mental health services who either accessed support, including inpatient and community mental health services, during the pandemic, or needed services but did not access them	Telephone and videoconferencing	Remote diagnostics and evaluation, accessibility to services, flexibility of services, care delivery mode via technology	a) sustaining capacity and enabling access to secondary mental health services; b) flexibility offered by remote care, particularly in the context of reduced access to face-to-face service provision. Disadvantages: c) consultations by telephone and video restricted therapeutic relationships compared with in-person contact, particularly where patients and care providers could not build on a bond already formed face to face
26	Watts 2020 [56]	RCT	Canada	Patients with generalized anxiety disorder	Videoconferencing	Accessibility to services, care delivery mode via technology	a) the use of video conferencing for telepsychotherapy did not negatively affect the establishment of quality working alliance in this sample of individuals with GAD; b) telepsychotherapy via videoconferencing may allow for the development of a significantly higher working alliance than conventional psychotherapy, at least from the perspective of clients suffering from GAD; c) clients rated the quality of the working alliance more positively than psychotherapists in the telepsychotherapy via videoconference condition;
27	Humer 2020 [57]	Quantitative	Austria	All licenced Austrian psychotherapists	Telephone, videoconferencing, email	Care delivery mode via technology	The experiences of psychotherapists with remote psychotherapy were better than their expectations but not totally comparable to face-to-face psychotherapy with personal contact
28	Feijt 2020 [58]	Qualitative	Netherlands	Practicing mental health care professionals	Video/telephone, chat sessions, e-mail, and e-health modules	Remote diagnostics and evaluation, accessibility to services, flexibility of services, care delivery mode via technology	a) the large majority of practitioners started using online tools on a daily basis; b) technological issues and limitations are frequently experienced, and practitioners feel insufficiently supported by their organizations in terms of technological support and hardware; c) miss the richness of nonverbal cues that are normally available in face-to-face sessions and important in establishing rapport with clients; d) Some clients lack the digital skills to work with the software, and sometimes the client’s home environment does not offer the required privacy needed for online treatment; e) beneficial in practical sense for care providers and patients; f) Distance created helps some patients as they become less inhibited in their expressions; g) higher adherence to treatment
29	Parikh 2021 [59]	Quantitative	US	Social workers, psychiatrists, residents, nurse practitioners/physician assistants	Videoconferencing	Privacy of care providers and patients, care delivery mode via technology	a) satisfaction and interest in continuing telepsychiatry was strikingly high; b) Just over two-thirds of providers reported that video visits allowed good interaction with patients as well as an effective approach to evaluate patients; c) Almost 70% of respondents felt their video visit appointments were the same or better than in-person appointments; d) nearly 80% of respondents were comfortable providing telepsychiatry
30	Pruitt 2022 [60]	Quantitative	US	Behavioural health and medical care providers who interact with individuals who may be at risk for suicide	Videoconferencing	Care delivery mode via technology	a) respondents are willing to provide suicide prevention services through telehealth; b) providers perceive suicide prevention services through telehealth as effective as face-to-face care
31	Sugarman 2021 [61]	Quantitative	US	Mental health/substance use disorder clinicians	Videoconferencing	Privacy of care providers and patients, flexibility of services, care delivery mode via technology	a) prevented sharp disruptions in care; b) clinical issues did not significantly impact patient care; c) clinicians generally agreed that that they could establish rapport with patients and treat their patients’ needs well through telehealth; d) however, agreement with these statements was consistently lower for group therapy, family therapy, and initial assessment visits; e) a decrease in no-shows and cancellations, f) and that they were able to see more patients, more frequently with telehealth care
**Mental health care providers and patients**							
32	Svistova 2022 [62]	Qualitative	US	Mental health service providers and representatives from Medicaid managed care organizations	Texts, videoconferencing, telephone	Accessibility to services, care delivery mode via technology	a) telehealth appeared to work well for youth and was reported to improve parental responsiveness and engagement in mental health care due to its convenience; b) decrease in appointment cancellations and no-show rates as one of the unintended consequences related to telehealth use; c) accessibility; d) engagement and involvement of family
33	Weiskittle 2022 [63]	Quantitative	US	Clinicians working with veterans	Telephone or videoconferencing	Flexibility of services, care delivery mode via technology	a) Veterans enjoyed the groups and desired to participate again in the future; b) technology challenges; c) The telephone modality was described as challenging when Veterans were more functionally impaired, but was preferred over having no intervention at all

There was a high level of diversity of study populations included. The majority of studies (*n* = 23) were undertaken with a wide range of service users and staff including: general population [33, 40], patients with psychiatric disorder [31, 35, 54], patients with social anxiety disorder [38, 46, 56], patients with substance abuse disorder [37, 48, 61], patients with serious mental health illnesses (SMI) [31, 34–36, 40–42, 44, 48, 50, 51, 58, 59], and the rest of 10 studies had a narrow and specific focus on homeless people [32], prison inmates [39], patients in psychiatric centres [42], veterans [43, 47, 63], patients of specific treatment units or clinics (a tertiary care anxiety disorders clinic [45], (The Salamanca Psychiatry Department [49], patients at risk of suicide [60] and acute psychiatric patients who require a higher level of care [53]. Several studies included various groups of patients or focus of care provision (for example: a study includes patients with SMI and substance abuse disorder [37].

### Changes in services reported

Most studies reported a transition of service delivery from in-person to a range of telehealth (*n* = 29) with only four studies reporting a hybrid method of service delivery. The most common teleconference method was through video calls (*n* = 15), multiple sources of contact through video, phone, texts, emails and electronic health consultations (*n* = 5), telephone (*n* = 5), both telephone and video calls (*n* = 4), face-to-face and phone or videoconferencing (*n* = 2), text messaging (*n* = 1) and use of immersive virtual reality reminiscence (*n* = 1).

The changes in mental health services were grouped into seven categories based on the description of key adjustments in service delivery and provision in response to COVID-19. They related to technological support when using or delivering care, accessibility and flexibility to services, remote diagnostics and assessment, privacy of patients and care providers, safety measures and operating hours of services (Table 2).

**Table 2:** Changes identified in mental health services table

Name of change	Studies	Changes reported	Examples (min. 2)
Care delivery via technology	*N* = 33 [31–63]	Service provision via technology was largely seen as a viable alternative to in-person mental health services. Here remote service provision was seen as a viable alternative to face-to-face care delivery	1. Such factors as efficiency in clinic’s work [40] clarity in service provision [45], speed of information delivery, real-time observations of patients’ home environment [58, 61], cost reduction were sent as factors supporting the use of remote service delivery. 2. Despite the mentioned technology advantages such as flexibility, accessibility and mobility of care providers, six studies highlighted that technology also meant that work became more isolated, and establishment of informal working relationships was difficult [32, 42, 44, 58–60]. 3. For those who did not know the patients well before the lockdown restrictions, building a good rapport with patients was challenging and sometimes impossible [31, 42, 58]
Accessibility to services	*N* = 25 [31–41, 43, 44, 46, 47, 49–56, 58, 62]	Accessibility of services means the ability of patients to receive medical care and engage with care providers when needed	1. Remote support was provided for those who resided in communal group accommodation (current and ex-substance users) which was a barrier for individuals to have enough space and place during remote meetings [32]. 2. Text messages were sent to patients which increased service users’ ability to receive medical care and information [33] 3. Laptops were provided to patients prior to treatment to ensure accessibility. Also, local police had contact details of patients to increase access to emergency services. [46] 4. Where the mental health services were provided in a closed unit due to COVID, it allowed patients to have uninterrupted treatment which was appreciated by patients [41]. 5. It was also reported that nursing care providers saw technology as a barrier to delivering effective care which also hindered the progress of care [52]
Flexibility of services	*N* = 17 [31, 32, 34–40, 42, 46, 53–55, 58, 61, 63]	Flexibility of services included adaptability of services according to external conditions/factors and to the needs of patients and service providers	1. Patients were offered a variety of ways for feedback and guidance including informational orientation sessions before therapy; diary keeping; brief feedback sessions with specialists [37] 2. When providing services to homeless population flexibility negatively impacted patients as they felt that they lost control of the help available, did not have any structure to support provision and lacked options to access it [32] 3. The flexibility was also reported as a negative aspect of change as it “may have also diminished the sanctity of treatment” which led to low attendance rates. [35] 3. During the pandemic people felt they lost control of their circumstances and were frustrated with being offered limited and no flexibility in options. One of the easiest ways to create a more positive experience of access was through giving individuals choices in their care [32]
Remote diagnostics and evaluation	*N* = 14 [31, 33, 37–40, 42, 44, 48, 49, 53–55, 58]	Remote diagnostics and evaluation included assessment of symptoms and physical and mental health conditions of a patient. This allowed to prescribe medication remotely, issue and plan treatment, evaluate environment of a patient	1. Remote diagnostics was particularly useful in functional appointments to renew medication prescriptions or complete quick health check-ups [31]; 2. Remote evaluation of symptoms enabled to recognise signs of acute substance withdrawal, improved evaluation of abuse or neglect and allowed assessment of home environment safety [40]; 3. Having remote assessments, patients experienced a more comfortable environment when staying at home, could express themselves more freely, save transportation time and costs, and/or requested less time off work [42]. 4. Legal hearings on involuntary medication use were held remotely [39]. 5. Patients reported that lack of face-to-face contact made it more challenging for care providers to identify—and help them recognise themselves—signs that their mental health was changing. [55] 6. Care providers argued that remote diagnostics reduced the ability to detect subtle body language, nonverbal cues and physical signs of a disease [42]. The diagnostics included pre-treatment self-assessment and a follow-up by clinicians, standardised measures in assessing treatment effectiveness, symptom evaluation and prescription evaluation. The remote assessment was more effective and robust when performed by video call than by telephone as it allowed inclusion of patients’ appearance, behaviours, movements and affect [40]
Privacy of care providers and patients	*N* = 13 [31, 32, 34, 35, 37, 39–42, 58–61]	Privacy of care providers and patients includes maintaining confidentiality of individuals and sharing information with only those who provide or receive medical care	1. Privacy of patients was difficult to maintain due to other family members at home. Sessions were held with patients in their closets, bathrooms, and cars, while other patients censored themselves due to lack of privacy and the potential of being overheard [35] 2. Treatment units had limited access to visitors which increased patients' privacy and confidentiality [34] 2. Participants' confidentiality was maintained whilst participants were asked to engage in sessions in a quiet, private room. This was not always possible so the privacy of patients was jeopardised [37]; 5. In prison settings correctional officers were positioned outside the closed door of a clinic room to maintain confidentiality [39]
Safety	*N* = 6 [32, 37–39, 44, 60]	Safety aims to prevent and reduce risks, errors and harm that occur to patients during provision of health care	1. In prison settings, safety protocols were developed to ensure patients' safety and sessions were held near patients’ housing units to reduce transfers and reduce congregation in waiting areas [39]; 2. In marginalised groups such as homeless people safety standards could not be maintained due to physical restrictions of care providers (such as high presence of drugs) were a source of frustration and led to subsequent care avoidance [32]
Operating hours of service provision	*N* = 4 [32, 36, 39, 49]	Working hours of face to face or remote service provision have been increased or reduced depending on various factors and conditions	1. Hours of service provision for supporting homeless people in substance use have been significantly reduced to support being available only between 9am to 5 pm. This often led to frustration among participants as they could not access care when they needed it the most and felt that there was a “brick wall” to access support for [32]. 2. Introduced single telephone line working 24/7; new introduced rota for emergency [36] 3. When patients were based in treatment centres (patients in prison) service provision often started from a point of physically escorting a patient to service. With it being remote, service provision reduction was related to decrease of custody care providers’ time to escort patients to treatment units which resulted in a more effective use of care providers’s time [39]

The changes in services in this study are found to focus on the procedural mechanisms of care delivery which influenced ways of service delivery (from face-to-face care delivery to telehealth or mixed), longevity of service delivery (reduction or increase of operating hours), characteristics of service delivery (flexibility/accessibility/privacy/safety). Despite the changes often being an ad-hoc response to lockdown restrictions, it produced diverse and diametrical impacts on both service users and staff. Studies have reported mixed experiences of receiving care remotely in a diverse range of service users. For example, service users who were homeless could not access the services and did not feel that their safety or privacy were maintained when using the services remotely [32]. The existing pre-pandemic research did not identify any specific groups of patients who experienced deterioration in access of telehealth and broadly referred to them as patients who live in isolated areas or those “wanting total anonymity for personal reasons” [64]. Conversely, new emergency contact line which was available 24/7 enabled more accessibility in service provision [36]. Combined with a variety of clinical settings (e.g. inpatient or outpatient care, closed units such as prison settings), models of service delivery (e.g. intensive compulsory therapy, optional regular text messages, voluntary group therapy), diversity of characteristics and sociodemographic factors of service users and staff specialities, evidence has been limited in the effectiveness and feasiblity of remote care implemented throughout the current health system, which is consistent with mixed impacts identified in this review. The existing evidence on use telemental health in pre-pandemic settings goes in line with some of our findings. Accessibility is found as the central feature of telemental health for the predominant number of patients which is supported by high satisfactory rates [64]. Among other changes, the existing literature reported cost implications [64] which was not identified in the current review.

### Outcomes reported

Ten outcomes domains were reported including (1) care access; (2) satisfaction with telehealth; (3) comparability of telehealth with face-to-face care; (4) treatment effectiveness; (5) continuity of care; (6) relationships between patients and care providers (includes relationships and interactions between patients and care providers); (7) remote detection and diagnostics in patients; (8) privacy; (9) treatment length and (10) work-life balance. The most common three outcome areas reported in the included studies were access to services, satisfaction with telehealth and comparability of telehealth with face-to-face care. Table 3 demonstrates a list of outcomes reported followed by examples of positive and negative outcomes.

**Table 3:** Outcomes Identified after changes implemented in mental health services table

Name of Outcome	Studies	Outcomes Reported	Examples
Care access (Includes Care access and Stress management when accessing care)	*N* = 21 [care access, *n* = 19 [31–33, 35–39, 42–44, 46, 49, 51, 55, 56, 58, 61, 62]; stress management, *n* = 9 [35, 38–40, 42, 46, 50, 51, 62]	Care access was one of the most prominent outcomes. It included removing barriers to accessing care such as time, money spent on travelling to and from a clinic, physical difficulty of travelling, safety issues and waiting times in clinics	1. By removing barriers such as time and money spent on travelling to and from clinic, patients and care providers were able to accommodate treatment considering individual needs [46]
Satisfaction with telehealth	*N* = 21 [26–33, 37, 38, 41–44, 46–51, 56, 59]	Studies reported patients’ and care providers' satisfaction with care delivered in a remote format (patients = 15 studies; care providers = 6 studies) where both patients and care providers rated their overall assessment of mental health services delivered via telehealth	1. Twelve studies demonstrated high satisfaction rates among patients and care providers highlighting that the care delivered was of appropriate standard followed by positive overall experiences [31, 37, 39–41, 52, 56, 58–62] 2. Nine studies reported heterogenous views and experiences of patients and care providers showing no significant differences in experiences of using care pre-pandemic and during the pandemic to mixed satisfaction rates depending on individual circumstances [35, 38, 42, 44, 49, 53–55, 63]. None of the included studies reported complete dissatisfaction with telehealth
Comparability of telehealth with face-to-face care	*N* = 15 [32, 38, 43, 45, 47–49, 51–54, 57, 59, 60, 62]	Studies reported a comparison between face-to-face services and telehealth. Comparative analysis was based on patients and/or care providers’ experiences, personal observations and views, effectiveness results and other factors	1. Veterans who attended an initial PC-MHI mental health visit via telehealth were less likely to receive same-day primary care compared to veterans who initiated care in person [43]; 2. Effectiveness of remote iVR reminiscence may be comparable to that of face-to-face iVR reminiscence [52]
Treatment effectiveness	*N* = 13 [31, 33, 37, 38, 45–47, 49, 52–54, 60]	Treatment effectiveness refers to success in treatment outcomes of mental health services or positive results during treatment	1. Four studies reported that telehealth was effective in care delivery and treatment and led to improvement of patients’ symptoms [31, 33, 46, 60]; 2. A small but significant positive effect of face-to-face treatment on reduction in symptom severity over time, relative to videoconference treatment [45]
Continuity of care	*N* = 12 [34, 35, 40, 42, 45, 47–49, 53, 54, 58, 62]	Continuity of care included patients remaining in care without dropping out of treatment for longer	1. Significantly more sessions were attended by participants in the videoconference versus face-to-face GAD groups [45] 2. A decrease in appointment cancellations and no-show rates is one of the unintended consequences related to telehealth use [62]
Relationships between patients and care providers (includes relationships and interactions between patients and care providers)	*N* = 9 [31, 32, 42, 49, 55, 56, 58, 61, 63, 65]	Experiences of relationships between patients and care providers were mixed and ranged from improved and well-established rapport between patients and clinicians to disconnected and impersonal care experiences	1. The positive experiences described increased frequency of contacts and strengthened contact ties between patients and care providers as a response to patient’s needs to ensure continuity of care [44]. 2. The introduction of additional assistance and support has been well received by patients. The latter was often related to care provider turnover which potentially deteriorated due to COVID-19. In the new realms of more disjointed care patients found a need to repeat their stories which often made them “relive” that experience over again [25]
Remote detection and diagnostics in patients	*N* = 7 [31, 33, 37–40, 58]	Remote detection and diagnostics in patients include assessment of patients’ symptoms, conditions or overall state by telehealth methods including telephone calls, online platforms, text messages	1. In studies where patients had access to technology required, assessment was seen as a positive outcome as it allowed patients to stay relaxed in a comfortable environment of their homes whereas care providers were able to assess their home environment safety, detect and evaluate signs of abuse and neglect, allow for evaluation of physical symptoms, including signs of acute substance withdrawal [37, 46]. 2. The self-disclosure of patients was reported as an important factor which brought new insights into assessment and examination [28]
Privacy	*N* = 5 [31, 32, 35, 44, 60]	Privacy regulations changed in order to maintain confidentiality and anonymity requirements of both patients and care providers in telehealth	1. In five studies, privacy regulations were difficult to follow due to lack of private space among patients (in a study by Abdullah et al. [33], patients were reported to have sessions in a closet, bathroom and car). 2. Lack of private space for telehealth was sometimes addressed by using text services as an alternative to avoid a pause treatment [29]
Treatment length	*N* = 4 [38, 45, 53, 54]	Studies highlighted that the number of days in completing a course of treatment was increased in comparison to face-to-face treatment as telehealth was often more time-consuming than in-person therapy	1. Although remote therapy was reported to be more demanding and tiring for patients [28] in a study by Bulkes et al. [46] patients in telehealth attended treatment for six hours per five days a week whereas patients in in-person treatment completed three hours per five days per week which in total meant that patients receiving remote treatment stayed 2.8 days longer in treatment than patients seen in person
Work-life balance	*N* = 1 [59]	Work-life balance is related to experiences of keeping personal life and work separate. This outcome was experienced by care providers	1. One study reported that due to difficulty of maintaining privacy regulations, care providers struggled to separate personal life from work [27]

### Impact of changes

**Fig. 2:**
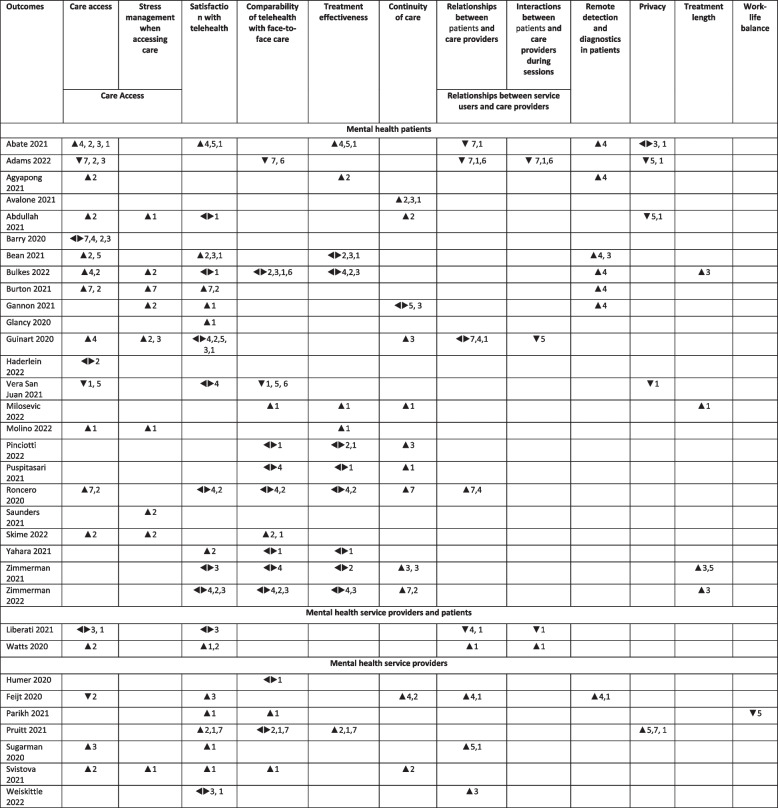
Effect direction plot of impact of changes in mental health service delivery on care providers and patients [31–63]

The effect direction plot (Fig. 2) was developed to visualise how the changes in mental health services impacted the experiences and outcomes reported in the included studies.

The effect direction plot illustrates a diversity of outcomes and often diametrically different experiences. Although the impact and experiences were very mixed across all of the included studies, there were two categories which demonstrated solemnly positive impact. It included improvement in stress management when accessing care and positive experiences of remote detection and diagnostics in patients.

Changes in flexibility, accessibility, operating hours of service delivery and methods of care delivery improved care access and reduced stress in accessing care [31, 33, 35, 37–40, 42, 46, 49–51, 56, 61, 62]. This was driven by four changes in service delivery including increased flexibility of service delivery, removing barriers to accessing care, positive changes in operating hours and care delivery via technology. Patients reported easier access (for example: [31, 35] less congested waiting rooms [39] and an increased likelihood of receiving same-day primary care compared to before the pandemic [43]. Appreciation of receiving support along with reducing risks of getting COVID-19 was also seen as an important feature of remote accessibility.

Remote diagnostics and assessment were reported in three studies where patients experienced improvement in accessing assessment services [31, 36, 42].

Conversely, three studies [32, 44, 58] reported reduced hours of service provision, lack of private space for meetings, absence of appropriate equipment and internet connection, and lack of IT skills, resulting in difficulties in receiving treatment. Although support was provided such as IT training and equipment provision, it was not always perceived as inclusive and feasible (for example: homeless [27], veterans [43] and elderly [52] did not have any access or skills to use technology [32]. It was also highlighted that Women and Black veterans were less represented among telehealth services when compared to in-person appointments [43].

Twelve papers found positive outcomes in patients’ continuity of care and extended length of care use [34, 35, 38, 42, 45, 47–49, 53, 54, 58, 62]. Seven studies related positive outcomes of remaining in care to improved accessibility to care [34, 35, 47, 53, 54, 58, 62] including technology-based care delivery and flexibility of care by both care users and care providers [58, 62].

Patients and clinicians were able to increase hours of care provision due to improved capacity for delivering mental health services [38, 45, 53, 54].

Eight studies showed no significant changes in treatment outcomes between face-to-face and remote care delivery [37, 38, 47–49, 52–54]. Five studies reported that remote diagnostics and evaluation, accessibility to services, privacy of care providers, safety of patients and care delivery mode via technology contributed to improvements in patient’s mental well-being [31, 33, 45, 46, 60].

Relationships between patients and care providers were influenced by changes in the mode of care delivery. Five studies reported negative outcomes of having remote care due to the transition to telehealth [31, 32, 42, 55, 56]. Care providers reported that changes in service delivery mode together with changes in privacy and remote diagnostics positively impacted their relationships with patients and were seen as a comfortable way of providing care [58, 59, 61, 63]. An included study [59] found that 70% of service providers felt that video appointments were the same or even better than in-person appointments.

Remote diagnostics and assessment were reported to be an effective approach to evaluate patients’ conditions [31, 33, 37–40, 58, 59, 66]. In addition to standardised assessment procedures, evaluation could also include such beneficial factors as real-time observations of home environment of a patient. As a negative impact, a fear of missing subtle signs of body language, nonverbal cues or physical signs of disease was seen as a possible barrier to using remote diagnostics [67].

A small number of studies reported that the privacy of patients and care providers was impacted by care delivered remotely. Lack of private and quiet spaces for an appointment, confidentiality challenges and inability to identify acute stress and agitation were all seen as barriers to maintaining private and safe care provision. One study reported improved experiences in privacy among care providers due to the transition to telehealth [32]. A combination of changes in privacy, safety and mode of care delivery all contributed positively privacy of patients. Three studies with patients [32, 35, 44] highlighted that changes in services delivered remotely jeopardised patients’ privacy due to the lack of private space for receiving care for patients.

Twenty-one studies [31, 35, 37–42, 44, 49, 52–63] reported patients’ and care providers satisfaction with care delivered in a remote format (patients = 15 studies; care providers = 6 studies). Twelve studies demonstrated high satisfaction rates among patients and care providers highlighting that the care delivered was of appropriate standard followed by positive overall experiences [31, 37, 39–41, 52, 56, 58–62]. Changes in service delivery mode via technology, accessibility and flexibility contributed to patients’ care satisfaction. Nine studies reported no differences in mixed satisfaction in experiences compared to pre-pandemic depending on individual circumstances.

Fourteen studies reported their assessment of service comparability [32, 38, 45, 47–49, 51–54, 57, 59, 60, 62]. Four reported preferences for telehealth compared to face-to-face provision [45, 51, 59, 62] due to remote care delivery method and accessibility to services. Nine studies suggested no difference [38, 47–49, 52–54, 57], and one study showed a preference for face-to-face care [32].

## Discussion

### Key findings

As a response to COVID-19, mental health services faced immediate challenges and opportunities to adapt the delivery of service provision underpinned by the need of increased physical distancing [68]. This often included telehealth referring to telemental health, telepsychiatry, teletherapy, or telepsychology [68]. Although telehealth has been in use for several decades, it has often been optional and has never been followed by a context of pressing and increased need for mental health services from wider population.

In the context of national reliance on mental health services’ transition to new safe formats of care delivery,, the review aimed to synthesise the changes to mental health services in response to COVID-19 and the impact of those changes. It identified changes to service delivery mainly from face-to-face provision to telehealth formats, primarily delivered through telecommunication technologies and videoconferencing supported by phone calls, text messages and hybrid modes of service provision. The service users in the included studies presented a range of population groups from broad catergories (general population, individuals with MCI or SMI) to narrow groups (veterans, prison inmates, eldery, homeless people, people at risk of suicide or at higher level of care). The existing research found that telemental health was predominantly used by women [69]. This has not been identified in our review due to lack of information on gender in included samples.

During a significant shift to telehealth the key changes in services included the transition to remote forms of care followed by technical aspects of using equipment among different patient populations, accessibility and flexibility of services, practicalities of diagnosing mental health conditions remotely, safety measures and privacy of remote services. Accessibility and flexibility were often identified as positive changes by a broad range of service users and care providers. The accessibility issue has also been evidenced in pre-pandemic use of telehealth where treatment facilities would otherwise be not available for underserved areas [70]. For some specific groups of patients (homeless people, current and ex-substance users) who heavily relied on mental health services providing space for therapy, accessibility was jeopardised by limited availability of safe and private space for remote meetings.

Where the operating hours of service delivery were increased either by introducing a new on-call member of care providers or opening a 24/7 emergency phone line, patients were also satisfied by the change. Conversely, in one patients’ group of homeless people operating hours were reduced which had a negative impact on their treatment. Implementation of remote mental health services was hindered by a series of barriers including identification of appropriate equipment, use of suitable applications and identification of telehealth strategies which would be patient-friendly and cost-effective. All of the mentioned features of telehealth require additional education for both patients and care providers which is also evident in other research [71–75]. Where remote diagnostics was introduced, it appeared as a useful element to maintain assessment of patients’ state and conditions whilst following COVID-19 lockdown regulations. Remote assessment was particularly effective in advancing severe mental illness including schizophrenia-spectrum disorders [76]. Our review demonstrates a variety of different patient groups including the general population who had digital evaluation applied successfully. Although changes in privacy and safety were rarely mentioned in included studies, they caused a lot of concern among both patients and care providers. It was suggested that specifics of maintaining privacy and safety during telehealth required training, awareness and knowledge which was not often possible due to limited resources and time.

The review demonstrated that experiences and outcomes for patients and care providers were mixed and influenced by the telehealth changes identified. Experiences related to care access, care effectiveness, continuity of care use, relationships between care providers and patients, privacy and remote diagnostics were reported. The strong interconnections between telemental health changes and their impact on patients’ and service providers’ experiences of care were also echoed in pre-, during and post- pandemic studies demonstrating varied outcomes [68, 74]. For example, the previous research found that telephone services were the most affordable and easily accessible form of care in comparison with other ways including face-to-face services [68]. However, the study also indicated strong preference of patients to use videoconferencing over telephone-based services which raised a dilemma of most effective service modality. In contrast our review did not identify clear connections of service mode and experiences of accessibility of patients. Instead, access to care was shaped and supported by changes in flexibility, accessibility, operating hours of service delivery and digital methods of care delivery and was improved in nearly half of the included studies. This validates other studies [77, 78] demonstrating that accessibility and flexibility are significant cues to attend telehealth treatment despite the absence of face-to-face appointments. Not only were opportunities to reach care affected, stress levels of patients were also impacted. Where mental health services could not accommodate easy access to treatment and its round-the-clock availability and had to reduce hours of service provision, patients reported negative mental well-being.

The evidence on mental health interventions and service changes has focused on increasing attendance and reducing non-attendance of patients [79]. This was echoed in this review that changes in improved access and flexibility of care were able to accommodate extended treatment length and reduced the number of dropouts from care.

Mixed interpersonal relationships between patients and care providers were identified by a rapid shift to telehealth. The existing pre-pandemic research suggested that the relationships between patients and care providers need to maintain a subtle balance between in-person and remote interactions to avoid risk of losing and openness [80]. In contrast to gradual transition to telehealth [81], a critical factor in a balanced care provision between in-person and remote care, a rapid shift to virtualization of both patients and care providers identified in this review subsequently led to some negative impact on patient-care provider relationships.

Positive outcomes in relationships which were reported by care providers were associated with accessible and regular appointments often easier to achieve in digital care as opposed to face-to-face provision. Conversely, due to care providers’ turnover patients experienced disjoined care. The pre-pandemic research reported that telehealth was “manageable” for patients when they received support from a care team they previously met face-to-face [82]. Our findings showed that rapid changes in staff in delivering telehealth often resulted in care provision by someone a patient did not know or met, leading to discontinuity of care.

Telemental health was considered to be “unquestionably effective” across many population [64] pre-pandemic and consistently useful in reduction of severe sysmptoms in patients with SMI [83]. The more recent research focusing on effectiveness of telemental health referred it as “not inferior to face-to-face services” [84] and found it to be a feasible mode of treatment and equally acceptable when compared with face-to-face services [68, 85–87]. In this review, although the predominant number of studies suggests that remote mental health care is a viable alternative to face-to-face service delivery with associated improved treatment outcomes we cannot draw the conclusion that telemental health is a desiarable form of mental health services due to heterogenious accessibility, privacy, safety and flexibility needs of patients. By synthesising the available evidence and a mixed range of population groups, patients’ mental health conditions, experiences and treatment arrangements in the included studies, it is important to consider the individual circumstances and patients’ needs when telehealth is applied.

### Limitations

This rapid review was designed and reported following recommendations by the Cochrane guidance for rapid reviews. A review protocol was designed and published [19]. A rapid yet thorough search of multiple databases was conducted. Despite the rigorous search, screening, and analysis process, we acknowledge the existence of some limitations of this study. Firstly, grey literature was not searched which may provide an important forum for disseminating studies that might otherwise not be disseminated. However we aimed to draw on conclusions based at rigorously designed and conducted studies but no all grey literature is subject to a similarly rigorous pre-publication review process [88]. Second, the selection criteria for this review centred on studies in English and limited to OECD countries to ensure a degree of commonality in context. In addition, a formal risk of bias assessment was not undertaken due to the design being a rapid review. However, this is the first rapid review addressing what and how mental health service changes impacted patients’ and care providers' outcomes during COVID-1.

## Conclusion

In this rapid review, we have reported changes in mental health services and patients and care providers’ outcomes which occurred in response to COVID-19 restrictions. A key finding of the review is the interconnection between changes in services and outcomes of patients and care providers. Changes in access to care, flexibility, hours of service provision and remote forms of telehealth in response to COVID-19 in mental health services could affect health outcomes and care experiences. Telehealth was perceived as an equally feasible mode of service delivery compared to face-to-face care and is argued to augment mental health care provision. A certain focus needs to be put on privacy regulations enabling patients to find space and time for appointments, use of technology, remote diagnostics and development of techniques which would identify nonverbal signs and cues and finally establishment of trusting and lasting relationships between service providers and patients.

### Supplementary Information

**Additional file 1.**

## Data Availability

All data generated or analysed during this study are included in this published article [and its Supplementary information files]. For any queries/requests related to data please contact the corresponding author [Evgenia Stepanova, email: evgenia.stepanova2@newcastle.ac.uk], upon reasonable request.
